# The Role of the IGF-1 Signaling Cascade in Muscle Protein Synthesis and Anabolic Resistance in Aging Skeletal Muscle

**DOI:** 10.3389/fnut.2019.00146

**Published:** 2019-09-10

**Authors:** Richie D. Barclay, Nicholas A. Burd, Christopher Tyler, Neale A. Tillin, Richard W. Mackenzie

**Affiliations:** ^1^Department of Life Sciences, University of Roehampton, London, United Kingdom; ^2^Division of Nutritional Sciences, Department of Kinesiology and Community Health, University of Illinois, Urbana, IL, United States

**Keywords:** IP6K1, sarcopenia, aging, protein, resistance exercise, anabolic resistance, Akt, mTOR

## Abstract

Sarcopenia is defined as the combined loss of skeletal muscle strength, function, and/or mass with aging. This degenerative loss of muscle mass is associated with poor quality of life and early mortality humans. The loss of muscle mass occurs due to acute changes in daily muscle net protein balance (NPB). It is generally believed a poor NPB occurs due to reduced muscle protein synthetic responses to exercise, dietary amino acid availability, or an insensitivity of insulin to suppress breakdown. Hence, aging muscles appear to be resistant to the anabolic action of exercise and protein (amino acids or hormonal) when compared to their younger counterparts. The mechanisms that underpin anabolic resistance to anabolic stimuli (protein and resistance exercise) are multifactorial and may be partly driven by poor lifestyle choices (increased sedentary time and reduced dietary protein intake) as well as an inherent dysregulated mechanism in old muscles irrespective of the environmental stimuli. The insulin like growth factor 1 (IGF-1), Akt /Protein Kinase B and mechanistic target of rapamycin (mTOR) pathway is the primary driver between mechanical contraction and protein synthesis and may be a site of dysregulation between old and younger people. Therefore, our review aims to describe and summarize the differences seen in older muscle in this pathway in response to resistance exercise (RE) and describe approaches that researchers have sought out to maximize the response in muscle. Furthermore, this review will present the hypothesis that inositol hexakisphosphate kinase 1 (IP6K1) may be implicated in IGF-1 signaling and thus sarcopenia, based on recent evidence that IGF-1 and insulin share some intracellular bound signaling events and that IP6K1 has been implicated in skeletal muscle insulin resistance.

## Setting the Scene

Sarcopenia is defined as the combined loss of skeletal muscle mass, function and strength (1) and it can progress at a rate of approximately 0.8% skeletal muscle loss per year from the 5th decade in adult life (2). Sarcopenia is diagnosed using a battery of clinical assessments and globally effects 1 in 10 adults above the age of 60 (3). Given that sarcopenia costs the American Health

Service an estimated $18.5 billion annually (4) and the United Kingdom's National Health Service £4592 for 11% of the total aging population which costs £2.5 billion annually (5), it is important that we develop a greater understanding of the cellular pathways that drive this disease and how interventions such as exercise and dietary protein are used to delay this processes implicated in its progression.

Human skeletal muscle is of high plasticity and is in a constant state of remodeling. Skeletal muscle remodeling occurs due to the dynamic balance between muscle protein synthesis (MPS) and muscle protein breakdown rates (MPB). The daily difference between MPS and MPB defines net protein balance (NPB), which is a key regulator of overall skeletal muscle mass. A positive NPB is generally indicative of a positive remodeling response that can be hypertrophic [i.e., increase fiber cross sectional area (6)] or non-hypertrophic [i.e., increased metabolic quality (7, 8)] in nature, whereas a reduced NPB reflects an overt phenotype being negative by inducing a loss of muscle mass or poor metabolic

quality (9). Changes in MPB are small in normal aging, whilst changes in MPS seem to be larger in amplitude and more obvious in response to the main anabolic stimuli to muscle tissue. As such, the measurement of MPS is the primary focus in human metabolic research (10).

**Figure 1:**
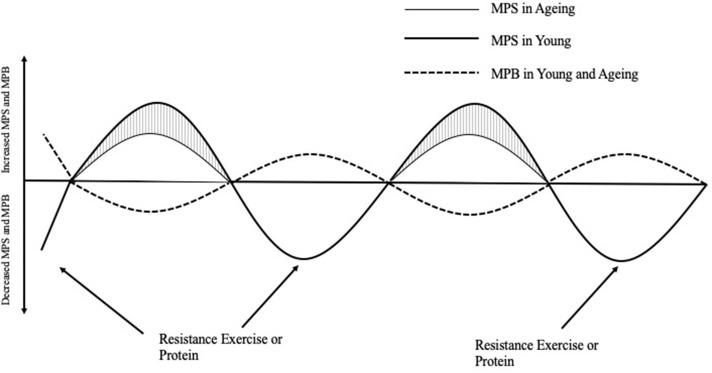
The response of muscle protein synthesis (MPS) and breakdown (MPB) on net protein balance after acute resistance exercise or protein ingestion in young and aging populations [Adapted from Breen and Phillips (12)]. In the morning after an overnight fast, muscle protein breakdown exceeds muscle protein synthesis such that net protein balance is negative. After a bout of resistance exercise or the ingestion of protein, young people respond greater in their myofibrillar protein synthesis response compared to aging people, which is appears to be the major attenuating factor to decreased NPB leading to skeletal muscle protein loss over time. MPS, Muscle protein synthesis; MPB, Muscle protein breakdown.

Protein ingestion stimulates an increase in MPS; however, a decrease in habitual physical activity, which is often observed with aging and/or injury, can induce anabolic resistance of MPS to protein ingestion (11) (Figure 1). For example, 7 days of unilateral leg immobilization in young men caused significant decreases in quadriceps cross sectional area (CSA) compared to the control limb and leucine supplementation did not reduce the loss in CSA (13). Similarly, 14 days immobilization in young men caused decreased CSA and an amino acid infusion in varying doses showed decreased post prandial MPS in the immobilized leg vs. the control limb (14). To reach the same myofibrillar protein synthetic response in muscle, older individuals need to consume more relative amounts of protein than younger individuals. *In vivo*, the stimulation of myofibrillar protein synthesis rates is dependent on intracellular molecular signaling pathways that become activated in response to extracellular cues. The mTOR complex 1 (mTORC1) pathway appears to play an important role in stimulating postprandial / post-exercise myofibrillar protein synthesis rates (15), and activation of this pathway can occur in two ways; firstly, through mechanical contraction (i.e., resistance exercise) which causes a release of skeletal muscle IGF-1 (8) and secondly via amino acid/protein intake (16). Furthermore, resistance exercise (RE) plus protein ingestion increases mTORC1 phosphorylation to a greater extent than protein or RE alone (17, 18), and likely intracellular redistribution of mTORC1 toward the sarcolemma as well (19).

As humans age, the muscle's ability to respond to both exercise and dietary protein diminishes leading to reduced NPB, and particularly in the myofibrillar protein fraction (11, 20, 21). The insulin like growth factor 1 (IGF-1), Akt/Protein Kinase B–mTOR pathway is acutely stimulated to promote ribosomal biogenesis and translation to form new myofibril proteins using these elongated ribosomes across the mRNA, which allows for the remodeling of skeletal muscle (22, 23). This occurs through amino acid sensed stimulation of phosphatidylinositol 3-phosphate (PtdIns3P) and localization of mTOR to the lysosome surface where mTOR positive lysosomes can translocate to the cell periphery which creates the most anabolic environment for myofibrillar remodeling (24).

Inositol hexakisphosphate kinase 1 (IP6K1) has recently been shown to inhibit Akt^308^ activity in IGF-1 stimulated hepatic cell lines (25) as well as skeletal muscle contraction having the ability to reverse this negative effect by reducing IP6K1 content (26). Normal IGF-1 signaling and Akt activity are vital for maintaining anabolic sensitivity (27). Taken together, it is hypothesized that IP6K1 may have a role in the onset of sarcopenia, and this review aims to discuss the possible role it has in anabolic resistance in aging skeletal muscle. Moreover, this review will describe and explain the molecular regulation of MPS and the role of RE and dietary protein has on the Akt-mTOR pathway in anabolic resistant, aging muscle.

## Molecular Regulation of Muscle Protein Synthesis (MPS) in Response to Exercise and Nutrition

MPS is regulated by an intrinsic cell signaling response which is activated by various external cues, such as dietary amino acids and RE. These cues drive the molecular regulation that augments MPS rates which facilitates the protein remodeling response in skeletal muscle, which is generally considered to be a hypertrophic response when studied during recovery from RE. The muscle protein remodeling response after endurance exercise is likely more aimed at non-hypertrophic remodeling. What is noteworthy is that it is still unclear how different anabolic signaling pathways coordinate the synthesis of specific muscle protein sub-fractions such as myofibrillar or mitochondrial proteins throughout the postprandial or post-exercise period. In addition, the time course of activation for these signaling transduction pathways is still unclear. The signaling response to RE and protein is described in detail below both in healthy and anabolic resistant phenotypes.

## Insulin Like Growth Factor-1 Activation

IGF-1, owing its name to high homogeneity to insulin, is a small peptide structurally bound by 70 amino acids (28). It is secreted by many tissues including the liver and skeletal muscle. Once IGF-1 enters the blood stream, there is not a specific tissue target (adipose, skeletal muscle, brain, cardiac muscle) although it is agreed that IGF-1 secreted from the liver (the largest contributor to circulating IGF-1), will not act on those tissues that have capabilities of producing the hormone themselves, such as skeletal muscle (29–33). At rest, circulating IGF-1 is relatively stable and young adults have significantly greater concentrations compared to older individuals (34). It has recently been suggested that large, but transient, increases in exercise-induced plasma IGF-1 concentrations are not required for activation of intracellular muscle signaling and the subsequent skeletal muscle adaptive response (35, 36). For example, Morton et al. (35) observed no relationship between circulating anabolic hormones (IGF-1) in plasma and strength gains in resistance trained young men after 12 weeks of low or high load RE. This suggests that intrinsic secretion of muscle IGF-1, not circulating plasma IGF-1, is a key determinant for switching on anabolic pathways (37). This is supported by Morton et al. (37) who suggested muscle androgen receptor content and not circulating hormones was associated with changes in lean body mass following 12 weeks of RE in previously trained men. In muscle, IGF-1 is stimulated by mechanical loading and contraction to which IGF receptor (IGFR) is activated in the cell to allow for membrane bound protein signaling pathways to become active. IGF-1 is secreted from muscle fibers into the extracellular matrix (ECM) to which it is bound by IGF binding proteins (IGFBPs). Given, the half-life of IGF-1 is just 5–10 min (38), these pools of IGFBPs must be local to the ECM (39–41). Upon binding to IGFBPs, IGF-1 activates its receptor to which intracellular signaling processes driving MPS can occur.

IGF-1 enters the cell via IGFR, it triggers phosphoinositide 3-kinase (P13-K) to generate hosphatidylinositol (4, 5)-bisphosphate (PIP2), leading to the production of hosphatidylinositol 3,4,5-trisphosphate (PIP3) (27, 42, 43). PIP3 is then free to bind to phosphoinositide-dependent kinase-1 (PDK1) which binds to the pleskstrin homology (PH) domain of Akt, allowing for translocation to the cell membrane preceding phosphorylation at Akt^308^ (27, 43).

## Akt-mTOR Signaling in Response to Protein and Resistance Exercise

Akt is a threonine and serine protein kinase which has three isoforms; Akt1, Akt2, and Akt3 (44). Akt1 has been linked to cell survival and may inhibit apoptosis (45). The same isoform is also implicated in MPS (46). Akt2 is the predominant form present in skeletal muscle and is heavily involved in glucose uptake and MPS via Akt^308^ and Akt^473^ (47) whilst Akt3 is vital for brain development and cell death (48). Diez et al. (49) described the activity of specific isoforms on Akt^308^ phosphorylation and despite Akt1 resulting in over half of total Akt expression, inhibition of Akt1 and Akt3 through use of shRNA techniques had no significant effect on total phosphorylated Akt^308^. In contrast to the little effect of Akt1 and Akt3 inhibition had on total phosphorylated Akt^308^, inhibition of Akt2 was related to reduction in total Akt and phosphorylated Akt^308^ in all conditions respective to control. Mutant mice lacking the Akt2 gene have significantly reduced total and phosphorylated Akt compared to mice lacking the Akt3 gene (49). Furthermore, Akt2 mutants had impaired glucose homeostasis, and growth deficiencies which may have been partially due to significantly blunted Ribosomal protein S6 kinase beta-1 (p70^s6k^) phosphorylation. Akt activation by mTOR complex 2 (mTORC2) uses PIP3, the product of P13K, to bind to the pleskstrin homology (PH) domain of mSin1 which localized mTORC2 to the cell membrane and relieves inhibition of mTOR which allows for Akt^473^ phosphorylation (50). Phosphorylation of both serine threonine residues are vital for maximal Akt activity and disruption of either site will have a knock-on effect downstream (51). In healthy phenotype, this mechanism occurs quickly and of high magnitude in response to RE (52) whilst this response is blunted in metabolically unhealthy or aging (53). Interference at any stage of this sequential reaction can have a profound effect on downstream signaling and metabolism. For example, inhibition of insulin receptor substrate 1 (IRS-1) in genetically modified mice had little effect on muscle atrophy, however when IGFR and IRS-1 are both disrupted, mice showed marked atrophy but had good glucose tolerance, measured by intravenous glucose tolerance test in gastrocnemius and soleus muscle which may have been due to increased glucose transporter 1 (GLUT-1) and glucose transporter 4 (GLUT-4) (42).

Downstream, Akt^308^ activates mTOR complex 1 (mTORC1) at mTOR^2448^ via the tuberous sclerosis complex (TSC). The TSC is phosphorylated by Akt which then disassociates the TSC from the small GTPase Ras homolog enriched in brain (Rheb) to localize to the lysosome where Rheb and mTORC1 can interact and phosphorylate mTORC1 (54). Similar to Akt, mTOR is a serine/threonine kinase and is made up of 5 components; Raptor, mLST, mTOR, PRAS40, and Deptor. All five of these components play a key role in mTORC1 signaling that subsequently results in MPS via downstream protein phosphorylation. Following RE and dietary protein ingestion, mTORC1 activity is upregulated which allows for subsequent membrane bound proteins to increase MPS (55). Phosphorylation and activity of mTORC1 at mTOR^2448^ and mTOR^2481^ are directly correlated with MPS (17, 56). mTORC^2481^ is a marker of intact mTORC2 and phosphorylation of this site is an indicator that the actin cytoskeleton and Akt^473^ are metabolically active (57). RE and dietary protein ingestion actively promotes skeletal muscle remodeling, at least partly, through acute activation of the Akt-mTORC1 pathway, which prevent MPB pathways such as autophagy (8, 27, 58, 59). Acute increases in Akt-mTORC1 signaling increase MPS to prevent breakdown in skeletal muscle, albeit MPS blunted in older humans compared to younger counterparts (8, 59). However, it has been established that targeted exercise prescription aimed at encouraging the recruitment of type II muscle fibers with the more volume (6 sets > 3 sets) can restore the post-exercise MPS response to a more youthful-state (60). To prevent negative protein balance and therefore muscle atrophy, RE and increased protein intake can be used as a tool to prevent progressive loss of muscle mass and function in aging adults (12, 21).

Downstream of mTORC1, two key proteins are activated to regulate muscle mass. Previous efforts have shown that mTORC1 signaling to p70^s6k^ and 4E-BP1 are both required for an optimal amount and quality of muscle mass during hypertrophic remodeling (61). In particular, 4E-BP1 phosphorylation can support hypertrophy, but without p70^s6k^ phosphorylation the quality of muscle is poor and leads to impaired force production due to formation of protein aggregates (61). In humans, it was demonstrated that p70^s6k^ phosphorylation 6 h post exercise is a strong predictor of skeletal muscle mass using a small sample size, which suggested that disruption of these anabolic signaling proteins can have a profound effect on muscle plasticity (62). However, Mitchell et al. (63) showed that the predictive nature of p70^s6k^ phosphorylation immediately resistance exercise for muscle mass gain is less apparent when using a larger sample size. Such a finding is consistent with the heterogeneity of skeletal muscle adaptive potential in humans (64, 65), and re-underlines the challenges associated with emphasizing the value of a single point snapshot of phosphorylation after exercise as the “holy grail” in terms of predicting the extent of skeletal muscle adaptations with progressive resistance exercise training in humans.

## Causes of Anabolic Resistance

There are several factors likely contributing toward the anabolic resistance of aging muscles to RE or protein ingestion (66), many of which have been highlighted above, including elevated mTORC1 phosphorylation in the post absorptive state (67). This systemic activation of mTORC1 in the post absorptive state inhibits autophagy in muscle, a protein breakdown pathway, which leads to the build-up of excess, unused ribosomes that have been synthesized as a result of mTORC1 phosphorylation and ultimately leading to reduced structural integrity of the ECM of muscle which causes insulin resistance (68, 69). It is thought that this is due to an insensitivity to anabolic stimuli whereby mTORC1 is elevated at rest and causing a systematic upregulation of MPS, similar to insulin resistance (67). A similar adaptation is seen in obese muscle that is resistant to anabolic stimuli (70); therefore, this suggests that a major difference between unhealthy and healthy muscle is post absorptive mTORC1 activity. TSC1 deficient mice, showing prolonged and chronic elevated mTORC1, developed late onset myopathy, showed vacuolated and basophilic fibers, intracellular inclusions, and abnormally large myonuclei. Furthermore, extracellular binding proteins were impaired in TSC1 deficient mice which may be an attenuating factor to myopathy. In older adults after an acute bout of RE, p70^s6k^ and 4E-BP1 do not increase to the same magnitude as young people (53). This decreased activation of key proteins may be caused by upstream signaling pathways being disrupted, however is it not possible to turnover new proteins without a distinct activation of p70^s6k^ and 4E-BP1. Drummond et al. (53) saw increases in 4E-BP1 phosphorylation at 1 and 6 h in young men however the increase was maintained for only 1 h in older adults. Kumar et al. (21) saw a similar effect in response to several different bouts of RE in a scaled protocol for one rep max percentage, older individuals had a significantly reduced FSR at all intensities compared to young and this response was coupled with a decreased p70^s6k^ phosphorylation. With the evidence above in mind, it is clear that elevated post absorptive mTORC1 activity is a negative adaptation, however the reasons to which this occurs are currently not known.

Recently a NAD+ dependent deacetylase called Sirtuin 1 (SIRT1) has gained some attention due to its emerging role in increased cell longevity (71). This protein is implicated in a number of pathways including cellular energy production, mitochondrial biogenesis via AMPK and PGC-1α while reducing inflammation through the inhibition of NF-kB (71–73). SIRT1 acts negatively on Akt and mTORC1 in the basal state, a finding previously shown to be a major attenuating factor to the normal response to anabolic stimuli (70). Furthermore, aging is associated with an increase in SIRT1 muscle content, however anabolic resistant skeletal muscle in aging populations display less SIRT1 vs. active counterparts, a finding that may contribute to increased post-absorptive Akt-mTORC1 activity (74, 75). Furthermore, caloric restriction also appears to increase SIRT1 muscle content, adding support for the combination of caloric restriction, RE, and leucine rich protein sources as a strategy for improving anabolic resistance in aging populations (71).

Habitual diet must be considered as a potential attenuating factor of sarcopenia given the reduced response older adults have to protein ingestion and the profound effect this has on skeletal muscle remodeling. Mediterranean diets rich in protein, fats and vegetables have been shown to reduce the risk of sarcopenia through its anti-inflammatory effects and high anti-oxidant content (76). Some research suggests that individuals consuming a diet high in protein also present with increased serum IGF-1 concentrations (77, 78). Yet more recently, older adults consuming a protein rich diet (1.4 g/kg/day) showed no difference in circulating IGF-1 levels vs. a low protein group (0.8 g/kg/day) (79). Whilst older adults clearly require greater quantities of protein to gain the desired MPS response, the evidence suggests that the IGF-1 pathway is not responsive to their habitual diet. A recent study on old mice (26 months old) suggested that increased expression of local skeletal muscle IGF-1 isoforms (IGF-1 Ea and IGF-1 Eb) counteracted sarcopenia, which included increased SIRT1 muscle content, autophagy and PGC-1α, without inducing any negative effects in other tissues compared to wild type mice (80). No study to the authors knowledge has investigated the local skeletal muscle isoforms of IGF-1 and the effect of acute or habitual diet on its concentration and secretion, and therefore requires exploration. Yet, as previously discussed, local skeletal muscle IGF-1 seems to play an important factor to skeletal muscle remodeling and that this effect is greater that any contribution to IGF-1 produced and secreted by other tissue types.

## Counteracting Anabolic Resistance With Nutrition and Exercise

**Figure 2:**
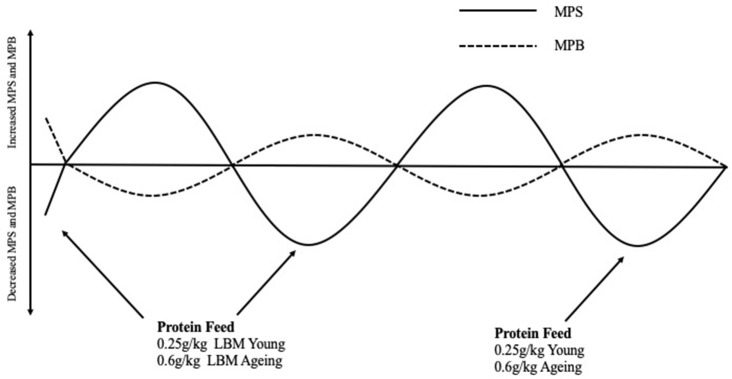
Normal fluctuations in Muscle Protein Synthesis and Muscle Protein Breakdown rates throughout the day in response to eating a protein containing meal and the effect this has on net protein balance. Protein Requirements to stimulate myofibrillar protein synthesis rates in young and aging populations are described in g/kg of lean body mass These protein meal requirements should be spread equally throughout the day (i.e., 4–5 meal times) to facilitate non-hypertrophic protein remodeling and counterbalance fasting-state protein losses that occurred in between meals (81). MPS, Muscle protein synthesis; MPB, Muscle protein breakdown; LBM, Lean body mass.

With the aim of counteracting anabolic resistance, studies have investigated how MPS can be maximized in older adults. Moore et al. (81) compared young and older adults myofibrillar protein synthetic response to graded intakes of whey protein and it was suggested that young and older adults could reach a similar response in their muscle however the quantity of protein required in g/kg lean body mass (LBM) in a single meal was far greater [0.6 g/kg LBM vs. 0.25 g/kg LBM (Figure 2)] in the older population. One explanation for this reduced ability of aging muscles to “sense” dietary amino acids in circulation is likely related to impairments in molecular signaling (e.g., decreased amino acid transports, elevated mTORC1, and IP6K1). Due to impaired molecular signaling intrinsic to muscle, older adults require greater amounts of leucine to have a similar response as younger individuals (81–83). To maximize MPS in aging adults, Kumar et al. (21) suggested that MPS was at its peak following 6 sets of 8 repetitions at 60% 1RM although this was significantly less compared to their young counterparts. This is important for the prescription of exercise protocols in research to ensure MPS is stimulated optimally and also important for exercise prescription in older adults aiming to prevent muscle atrophy and improve muscle function.

In summary, RE and higher protein diets have the ability to restore the anabolic sensitivity of MPS rates in aging adults, and either low or high load RE has the ability to stimulate a robust post-exercise muscle protein synthetic response throughout adult life. A sedentary lifestyle and reduced protein intake progressively cause a reduced response to anabolic stimuli (driven by blunted Akt-mTOR signaling), which leads to the progressive loss of muscle mass and function over time. Adults should engage with RE throughout their lives (2–3 times per week), and current evidence suggests that higher dietary protein intakes (0.6 g/kg LBM in each meal X 4–5 meal times) combined with modest caloric restriction is required to support a robust simulation in postprandial MPS rates and perhaps to maintain muscle mass and function with age. It is important to note, however, that a large-scale randomized clinical trial that combines exercise and protein intake manipulations (from RDA and beyond) is needed to confirm the value of eating protein in far excess of the RDA to support a more youthful phenotype with age. In addition, design of RE programme should consider adherence, motivation and enjoyment as these are the key factors for long term participation and ultimately skeletal muscle health.

## Possible Mechanism of Anabolic Resistance Involving Inositol Hexakisphosphate Kinase 1 (IP6K1)

**Figure 3:**
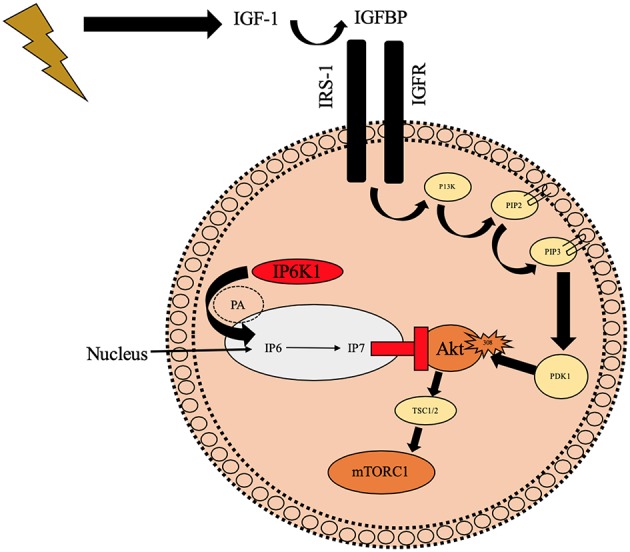
Schematic diagram illustrating the potential negative role of IP6K1 on Akt translocation to the cell membrane preventing phosphorylation of Akt^308^ which may reduce mTORC1. IP6K1 enters the nucleus via PA and it then synthesizes IP7 from IP6 which prevents Akt from translocating to the cell membrane and ultimately preventing Akt^308^ phosphorylation. IGFBP, Insulin like growth factor binding proteins; IGF-1, Insulin like growth factor-1; IP6K1, inositol hexakisphosphate kinase 1; IGFR, Insulin like growth factor receptor; IRS-1, Insulin receptor substrate 1; P13K, phosphoinositide 3-kinase; PIP2, hosphatidylinositol (4, 5)-bisphosphate; PIP3, hosphatidylinositol 3,4,5-trisphosphate; PDK1, phosphoinositide-dependent kinase-1; Akt, Protein kinase B; mTORC2, Mechanistic target of rapamycin; PA, Phosphotadic acid; IP6, inositol hexaphosphate; IP7, Inositol pyrophosphate; 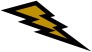 Illustrates contraction of skeletal muscle; 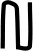 Illustrates binding/translocationto the cell membrane; 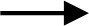 Illustrates activation; 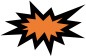 Illustrates phosphorylation; 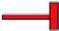 Illustrates binding to PH domain and downregulating Akt; 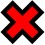 Illustrates preventing translocation to cell membrane.

Inositol hexakisphosphate kinase 1 (IP6K1) is a six carbon cylitol kinase which has recently been well documented in the attenuation of insulin resistance and type 2 diabetes (25, 26, 84–87). IP6K1 synthesizes IP6 to IP7 which binds to the PH domain of Akt (Figure 3), preventing translocation to the cell membrane (25). Inhibition of IP6K1 using N2-(m-Trifluorobenzyl), N6-(p-nitrobenzyl) purine (TNP) prevents diet induced obesity in mice through increased Akt activity (88). These mice maintained lean mass and had improved insulin sensitivity, reduced blood glucose, and insulin. Similarly, in Chakraborty et al. (25) study, mice that were treated with TNP had similar traits to the above mice in response to a high fat diet. They had healthy metabolic parameters (low serum insulin and blood glucose) and increased energy expenditure compared to wild type mice. Phosphorylation of Akt^308^ and p70^s6k^ was increased in TNP treated mice which increased glucose uptake and reduced fat mass whilst maintaining lean body mass, therefore having a positive effect on skeletal muscle remodeling, likely via mTORC1 and p70^s6k^ activity. In the same study, Chakraborty et al. (25) treated mouse embryonic fibroblasts with 100 mCi[^3^H]myoinositol for 3 days to inhibit inositol phosphates before stimulating with or without IGF-1. Graded concentrations of IGF-1 increased IP7 cell content in wild type cell lines vs. inositol knockout (-/-inositol) cells. In the -/-inositol cells, Akt^308^ and Akt^473^ activity increased with IGF-1 administration and more importantly for our hypotheses, p70^s6k^ activity was increased in the same way. The mTORC1 regulators TSC were also phosphorylated in IGF-1 treated -/-inositol cell lines compared to control which suggests IP6K1 could be implicated in Akt-mTORC1 signaling and therefore sarcopenia. Given this evidence it is conceivable that IP6K1 may have a role in the onset of anabolic resistance in aging phenotype via downregulated Akt, TSC and mTORC1.

Recently, our research group showed that IP6K1 was altered in adult (47 years ± 3) skeletal muscle in response to exercise (26). In this case, insulin resistant pre-diabetic individuals, whose IP6K1 was elevated at basal, were able to decrease the content in skeletal muscle following an acute high intensity interval training (HIIT) protocol (26). In this study, a HIIT intervention was superior compared to a continuous exercise protocol for increasing Akt^308^ activity and decreasing IP6K1 content. Given the findings of Chakraborty et al. (25), Naufahu et al. (26) and O'Neill et al. (42), it is hypothesized that elevated IP6K1 has a role in MPS via reduced Akt activity and physical inactivity. Habitual RE attenuates anabolic sensitivity via increased Akt and mTOR activity in response to anabolic stimuli (12, 89). Previous work has highlighted the negative role IP6K1 has on Akt activity whilst this negative effect is reversed with HIIT (26), thus we hypothesize that IP6K1 may be downregulated following RE in older adults.

To conclude, dysregulated Akt-mTOR signaling in response to RE and protein intake ingesting a current recommended intake (15 g per meal or 0.8 g protein/kg/day) is the primary driver of anabolic resistance and sarcopenia (90). Recent research has aimed at identifying strategies to maximize the response in the muscle in older individuals (21, 81), which include 0.6g/kg LBM protein in each meal and 60% 1RM for 6 sets of 8 repetitions. Thus far, characteristics of anabolic resistant muscle are known (i.e., elevated post absorptive mTORC1 phosphorylation, reduced SIRT1 muscle content, reduced structural integrity via reduced autophagy, and muscle atrophy); however, it is not clear why reduced protein intake and exercise cause these characteristics. With the evidence presented above, it is hypothesized that IP6K1 has a role in this diminished response to anabolic stimuli, similar to the onset of insulin resistance, and obesity. We are currently testing this hypothesis *in vitro* and *in vivo* at our University of Roehampton labs with the aim of disseminating our findings over the next 12 months.

## Author Contributions

RB was responsible for writing the manuscript. NB, NT, CT, and RM were all responsible for reviewing and contributing to the manuscript. RM was the senior author in the group.

### Conflict of Interest Statement

The authors declare that the research was conducted in the absence of any commercial or financial relationships that could be construed as a potential conflict of interest.

## Abbreviations

IP6K1: Inositol hexakisphosphate kinase 1
IGF-1: Insulin like growth factor-1
Akt: Protein kinase B
mTOR: Mechanistic target of rapamycin
mTORC1: Mechanistic target of rapamycin complex 1
SIRT1: Sirtuin 1
AMPK: 5′ AMP-activated protein kinase
Peroxisome proliferator-activated receptor-γ coactivator: PGC-1α
Nuclear factor kappa-light-chain-enhancer of activated B cells: NF-kB
PB: Net protein balance
RE: Resistance exercise
MPS: Muscle protein synthesis
MPB: Muscle protein balance
CSA: Cross sectional area
IGFR: Insulin like growth factor receptor
ECM: Extracellular matrix
IGFBP, Insulin like growth factor binding proteinsl PH: Pleckstrin homology
P13K: Phosphoinositide 3-kinase
PA: Phosphotadic acid
PDK1: Phosphoinositide-dependent kinase-1
PIP3, Phosphatidylinositol: 3,4,5-trisphosphate
PIP2: Phosphatidylinositol (4,5)-bisphosphate
mTORC2: Mechanistic target of rapamycin complex 2
IRS1: Insulin receptor substrate 1
GLUT-1: Glucose transporter-1
GLUT-4: Glucose transporter-4
TSC: Tuberous sclerosis complex
p70^s6k^: Ribosomal protein S6 kinase beta-1
4E-BP1: 4E Binding protein 1
FSR: Fractional synthesis rate
IRM: 1 Repetition maximum
IP6: Inositol hexophosphate
IP7: Inositol pyrophosphate
TNP, N2-(m-Trifluorobenzyl): N6-(p-nitrobenzyl)purine
HIIT: High intensity interval training.
